# Complete Genome Sequences of Identical *Zika virus* Isolates in a Nursing Mother and Her Infant

**DOI:** 10.1128/genomeA.00231-17

**Published:** 2017-04-27

**Authors:** Gabriela M. Blohm, John A. Lednicky, Marilianna Márquez, Sarah K. White, Julia C. Loeb, Carlos A. Pacheco, David J. Nolan, Taylor Paisie, Marco Salemi, Alfonso J. Rodríguez-Morales, J. Glenn Morris, Juliet R. C. Pulliam, Alejandra S. Carrillo, Juan D. Plaza, Alberto E. Paniz-Mondolfi

**Affiliations:** a Department of Biology, College of Liberal Arts and Sciences, University of Florida, Gainesville, Florida, USA; b Department of Pathology and Laboratory Medicine, Hospital Internacional Barquisimeto, Lara, Barquisimeto, Venezuela; c Emerging Pathogens Institute, University of Florida, Gainesville, Florida, USA; d Department of Environmental and Global Health, College of Public Health and Health Professions, University of Florida, Gainesville, Florida, USA; e Health Sciences Department, College of Medicine, Universidad Centroccidental Lisandro Alvarado, Barquisimeto, Lara, Venezuela; f Policlínica Barquisimeto, Barquisimeto, Lara, Venezuela; g Department of Pathology, Immunology, and Laboratory Medicine, College of Medicine, University of Florida, Gainesville, Florida, USA; h Public Health and Infection Research Group, Faculty of Health Sciences, Universidad Tecnológica de Pereira, Pereira, Colombia; i Division of Infectious Diseases and Global Health, Department of Medicine, College of Medicine, University of Florida, Gainesville, Florida, USA; j DST-NRF Centre of Excellence in Epidemiological Modelling and Analysis (SACEMA), Matieland, South Africa; k Health Sciences Department, College of Medicine, Universidad Nacional Experimental Francisco de Miranda, Punto Fijo, Falcon, Venezuela

## Abstract

Complete genome sequences were obtained for Zika viruses isolated from the breast milk of a Venezuelan patient and her child, who was exclusively breastfeeding at the time. These sequences are the first to be reported from a presumptive autochthonous postnatal transmission case from mother to child in Venezuela.

## GENOME ANNOUNCEMENT

*Zika virus* (ZIKV) is spreading widely in South and Central America, the Caribbean, and the Pacific (1). Although transmission of ZIKV is primarily mosquito-borne, direct transmission (e.g., through sexual contact and during pregnancy) is more common than previously expected (https://www.cdc.gov/zika/transmission/). We report here two complete genome sequences of ZIKV from two Venezuelan patients, where breastfeeding was the most likely mode of transmission.

The first isolate (VEN/UF-1/2016) is from the breast milk of a mother who developed symptoms of Zika fever (ZF) on 22 March 2016. The second isolate (VEN/UF-2/2016) is from the urine of her 5-month-old child who was exclusively breastfeeding and, interestingly, did not develop ZF. Breast milk, serum, and urine specimens were collected from the mother, and serum and urine specimens were collected from the child on 25 March 2016 at the Hospital Internacional Barquisimeto in Cabudare, Venezuela. All specimens tested positive for ZIKV genomic RNA (vRNA) by real-time PCR (RT-PCR). To determine whether the virus was infectious, aliquots of the specimens were inoculated onto LLC-MK2 cell cultures. Cytopathic effects (CPE) characteristic of ZIKV infection (2) were observed in all cell culture inoculations. To obtain sequencing templates, vRNA was extracted from the spent medium of cells inoculated with the mother’s milk or child’s urine using the QIAamp viral RNA minikit (Germantown, MD). Sanger Sequencing was completed using a genome walking strategy, as described previously (2). Briefly, cDNA was produced using AccuScript high-fidelity reverse transcriptase (Agilent Technologies, Santa Clara, CA) and sequence-specific primers. The resulting cDNA was amplified by PCR with Phusion polymerase (New England BioLabs) and gene-specific primers. The 5′ and 3′ ends of the viral genome were determined using a Rapid Amplification of cDNA Ends (RACE) kit (Life Technologies, Inc., Carlsbad, CA, USA). The sequences were assembled with Sequencher DNA sequence analysis software version 2.1 (Gene Codes, Ann Arbor, MI, USA). For phylogenetic analyses, ZIKV full-genome sequences were aligned using ClustalW (3) and BioEdit (4). The maximum likelihood phylogenetic tree was inferred from the full-genome alignment using the best fitting substitution model with IQ-TREE (http://www.cdc.gov/zika/transmission/) (5). Statistical robustness and reliability of the branching order within the tree were assessed by bootstrapping (1,000 replicates) and fast likelihood-based Shimodaira-Haswgawa (SH)-like probabilities (6) with IQ-TREE.

Full-genome comparison of the two ZIKV isolates revealed >99% identity between the two strains, with only two synonymous nucleotide substitutions at the third codon positions. The ZIKV sequences of the mother and child cluster with high bootstrap support (99%) within a larger clade of Colombian sequences. Both strains were different from the genomic sequences of ZIKV strains in the laboratory. The subjects of this report live in Barquisimeto, which is located along a major trade route between Colombia and Venezuela. The presence of infectious virus in the mother’s breast milk is consistent with the findings of other recent studies (7–10) and suggests that breastfeeding could be an additional mode of direct transmission for ZIKV. We report here the first complete genome sequences of ZIKV isolated from a clinical breast milk sample in a patient from Venezuela.

### Accession number(s).

Sequences have been deposited in GenBank under the accession numbers KX702400 (mother) and KX893855 (child).
